# A Comprehensive Review of Digestive Endoscopy-Associated Infections: Bacterial Pathogens, Host Susceptibility, and the Impact of Viral Hepatitis

**DOI:** 10.3390/microorganisms13092128

**Published:** 2025-09-11

**Authors:** Deniz Günșahin, Vasile Șandru, Gabriel Constantinescu, Mădălina Ilie, Teodor Cabel, Ramona Ștefania Popescu, Bogdan Silviu Ungureanu, Victor Daniel Miron, Gheorghe G. Balan, Diana Cotigă, Bogdan Miutescu, Gülşen Özkaya Şahin, Oana Săndulescu

**Affiliations:** 1 Faculty of Medicine, Carol Davila University of Medicine and Pharmacy, 050474 Bucharest, Romania; deniz.gunsahin@drd.umfcd.ro (D.G.);; 2 Department of Gastroenterology, Clinical Emergency Hospital of Bucharest, 105402 Bucharest, Romania; 3 National Institute for Infectious Diseases “Prof. Dr. Matei Balș”, 021105 Bucharest, Romania; 4 Gastroenterology Department, University of Medicine and Pharmacy of Craiova, 200349 Craiova, Romania; 5 Department of Gastroenterology, Faculty of Medicine, “Grigore T. Popa” University of Medicine and Pharmacy, 700115 Iași, Romania; 6 Emergency University Hospital, 050098 Bucharest, Romania; 7 Department of Gastroenterology and Hepatology, “Victor Babes” University of Medicine and Pharmacy, 300041 Timisoara, Romania; 8 Advanced Regional Research Center in Gastroenterology and Hepatology, “Victor Babes” University of Medicine and Pharmacy, 300041 Timisoara, Romania; 9 Department of Translational Medicine, Faculty of Medicine, Lund University, 20502 Malmö, Sweden; 10 Laboratory Medicine, Department of Clinical Microbiology, Skåne University Hospital, 22242 Lund, Sweden; 11 Academy of Romanian Scientists, 023993 Bucharest, Romania; 12 Infection Science Forum, 030175 Bucharest, Romania

**Keywords:** endoscopy-associated infections, MDR, ERCP, viral hepatitis, endoscopic ultrasound, antibiotic prophylaxis, endoscope reprocessing

## Abstract

Gastrointestinal (GI) interventional endoscopy has evolved into a cornerstone of modern gastroenterology, offering minimally invasive solutions for complex conditions. However, these procedures are not without risk, particularly with respect to post-procedural infections. While rare, such infections can have significant clinical consequences and are increasingly recognized as a public health concern. This narrative review provides a comprehensive overview of infections associated with GI endoscopy, focusing on transmission mechanisms, microbial agents involved, host susceptibility, preventive strategies, and diagnostic and therapeutic approaches. Infections following GI endoscopy remain infrequent but clinically significant, particularly in high-risk procedures such as endoscopic retrograde cholangiopancreatography and endoscopic ultrasound. Duodenoscopes represent a major vector for exogenous infection, often involving multidrug-resistant bacteria such as *Klebsiella pneumoniae*, *Pseudomonas aeruginosa*, and *Enterococcus* spp. Host-related factors increase the risk of infection. Risk factors associated with post-endoscopic infections include advanced age, male sex, non-white ethnicity, immunosuppression, presence of cholangiocarcinoma, autoimmune diseases, liver cirrhosis of viral and/or alcoholic etiology, chronic kidney disease, high-risk cardiac conditions, or chemotherapy. New reprocessing methods, such as ethylene oxide gas sterilization, automated endoscope reprocessors, and selective use of single-use endoscopes and duodenoscopes, may contribute to lowering infection rates. Greater awareness of infection risks, improved infection control practices, and adherence to updated guidelines are crucial for enhancing patient safety in digestive endoscopy. Multidisciplinary strategies, including surveillance, device innovation, and personalized risk assessment, are needed to address this evolving challenge.

## 1. Introduction

In recent years, interventional gastrointestinal (GI) endoscopy has undergone exponential growth in its capacity to manage a wide range of pathologies affecting the entire GI tract. Novel endoscopic techniques have been developed for the resection of polypoid lesions, the treatment of biliary and pancreatic diseases, and even for performing bariatric procedures. Many of these conditions were previously managed surgically; therefore, the transition from conventional surgery to endoscopic therapy represents a major advancement in the medical field, leading to reduced healthcare costs as well as improved morbidity and mortality outcomes [1,2,3]. However, interventional endoscopic techniques involve a range of invasive maneuvers that may be associated with various post-procedural infectious complications [4]. In addition, endoscope contamination is being increasingly reported worldwide, likely as a result of the growing use of interventional endoscopy, as well as advancements in microbiological diagnostic methods. The most frequently documented cases of infection transmission via endoscopy involve duodenoscopes, primarily due to their complex design, although infections associated with gastroscopes and colonoscopes have also been reported [5]. This issue is particularly significant given the widespread use of endoscopic procedures. In the United States alone, over 23 million endoscopic procedures are performed annually, including approximately 15 million colonoscopies, 7 million upper endoscopies, and 500,000 endoscopic retrograde cholangiopancreatographies (ERCPs) [6].

The incidence of GI endoscopy-associated infections varies depending on the type of procedure performed and patient-related factors, with the pooled overall rate reported at 0.2% [7]. The highest incidence has been reported in ERCPs performed for biliary obstruction, reaching up to 18% [8]. The infection rate after stent placement is high, ranging from 4.3% to 16%, with the benefit of prophylactic antibiotic therapy. In the case of endoscopic ultrasound (EUS), the incidence of post-EUS infections for the diagnostic evaluation of pancreatic cystic lesions ranges from 0.44% to 2%, regardless of the use of prophylactic antibiotic therapy [9].

A recent article reiterated that digestive endoscopy-associated infections remain an important concern, with multiple reported outbreaks, including those caused by multidrug-resistant (MDR) organisms, even after high-level disinfection (HLD). This review highlights two important issues: the need for post-procedural infection surveillance beyond 30 days and the inappropriate use of antibiotics due to false-positive microbiological results obtained from patients examined with already contaminated endoscopes [10].

Looking back at a 2018 report, infections transmitted through flexible endoscopes ranked among the top ten health technology hazards [11]. The most frequently encountered problems include contamination, incomplete sterilization, and inadequate drying of the working channel. These issues are especially common in used endoscopes, which often present surface defects, either microscopic or macroscopic, that facilitate biofilm formation and attachment [12].

The aim of this narrative review is to provide a comprehensive overview of the pathogens that can be transmitted through GI endoscopy, infection rates, current challenges and techniques in the reprocessing of flexible endoscopes, prevention strategies, and the use of prophylactic antibiotics in interventional endoscopy according to current guidelines, as well as key aspects in the diagnosis and management of post-endoscopic infections. This review serves as a valuable resource for both clinicians and researchers, offering up-to-date and relevant information while summarizing the most important data regarding infections associated with endoscopic procedures.

This narrative review was structured around a central research question (what are the current insights into the pathogens, risk factors, and prevention strategies for infections associated with digestive interventional endoscopy?) and organized into key thematic sections addressing the types of interventional endoscopy, mechanisms of infection transmission, implicated pathogens, risk factors, and prevention and management strategies. To identify the relevant literature, we conducted a search using PubMed, Web of Science, and Embase. We focused on articles published in the last decade but included older studies when particularly relevant. The selection was based on relevance, originality, and contribution to the understanding of endoscopy-associated infections. We used combinations of keywords and MeSH terms such as: “digestive endoscopy”, “endoscopy-associated infections”, “cross-infection”, “bacterial pathogens”, “viral hepatitis”, “healthcare-associated infections”, “risk factors”, “antibiotic prophylaxis”, “infection control”, and “post-endoscopic complications”. Additional references were retrieved by reviewing the bibliographies of selected papers and guidelines.

## 2. Types of Interventional Endoscopy of the Gastrointestinal Tract

GI endoscopy can be categorized into two main types: diagnostic and interventional. Both approaches can be applied to the upper and lower GI tract. Endoscopy emerged from the need to visualize the digestive tract through natural orifices, with its origins tracing back to antiquity. The introduction of flexible endoscopes and fiber optics has paved the way for increasingly complex endoscopic procedures capable of managing conditions that were once treated exclusively through surgical means [13]. The upper GI tract is examined using either forward-viewing gastroscopes or enteroscopes. Gastroscopes are most commonly employed to visualize the GI tract up to the second portion of the duodenum. Enteroscopes, which are thinner and longer than gastroscopes or colonoscopes, provide diagnostic and therapeutic access to the small bowel through an anterograde or retrograde approach. A distinct category is represented by side-viewing duodenoscopes, which are used in both ERCP and EUS. Their key features include the lateral positioning of the camera and the presence of an elevator mechanism, which plays a significant role in the pathogenesis of post-endoscopic infections [14].

Transient bacteremia may occur during endoscopic procedures, with specific risk rates associated with each type of procedure. However, this does not necessarily lead to clinical infection, as transient bacteremia can also arise during routine daily activities such as tooth brushing without any clinical consequences in the absence of relevant patient risk factors [4]. Clinically manifest infections depend on the type of procedure performed and the patient’s individual characteristics. Interventional endoscopic procedures carry a higher risk of infection compared to diagnostic procedures, as they involve the disruption of natural biological barriers and often require longer procedural times. The main interventional upper GI procedures include endoscopies performed for GI bleeding, which may involve esophageal variceal ligation or ulcer hemostasis. Additionally, biopsy of various GI lesions is also considered invasive and may result in clinically significant infections in certain patients. Colonoscopy and rectosigmoidoscopy are commonly used to explore the lower GI tract and range from purely diagnostic procedures to advanced resections such as endoscopic mucosal resection (EMR) and endoscopic submucosal dissection (ESD). Endoscopic stenting of the esophagus, stomach, small intestine, or recto-colonic segments are also invasive procedures. Frequently performed interventional procedures include ERCP and EUS combined with fine-needle aspiration (FNA) for cystic lesions or fine-needle biopsy (FNB) for solid tissue sampling, as well as percutaneous endoscopic gastrostomy (PEG) placement [15].

Contamination rates vary depending on the type of flexible endoscope used. Duodenoscopes, which are considered to be among the primary vectors for exogenous infection transmission, show contamination rates ranging from 0.3% to 30%, while the associated risk of infection in patients has been reported to range between 12% and 41% [5].

Hutfless et al. analyzed a cohort of over 800,000 ERCPs and reported a post-procedural infection rate of 3.5% at 7 days and 7.7% at 30 days. Identified risk factors for infection included emergency ERCP, pre-existing infections, chronic comorbidities, age over 65 years, male sex, and non-white ethnicity [16]. Although duodenoscopes are also used in EUS, the infectious risk remains significantly lower, with reported bacteremia rates ranging from 0% to 5.8%. It is important to emphasize that post-EUS bacteremia is generally transient and does not lead to clinical infection. However, drainage procedures for pancreatic or mediastinal cysts performed using FNA carry a higher risk, with reported infection rates between 0.4% and 0.9% [17].

In another study, Wang et al. evaluated the incidence of infections at 7 and 30 days following endoscopic procedures across six U.S. states. The post-procedural infection rate after a screening colonoscopy was 1.1‰, while for non-screening colonoscopies it was 1.6‰, and for upper GI endoscopies it reached 3.0‰ at 7 days. At 30 days, these rates increased to 4.0‰, 5.4‰, and 10.8‰, respectively. These rates also include complications related to general anesthesia and the risk of aspiration pneumonia [6].

In PEG, the most common infection is at the stoma site, with reported rates ranging from 4% to 30%. This occurs despite the use of antibiotic prophylaxis, which, according to some studies, does not appear to significantly reduce the risk [18,19].

For ESD, data on infectious complications are limited. However, post-procedural fever has been reported in 46.7% to 58.3% of cases, without clear evidence of active infection, and is often attributed to alternative mechanisms such as post-coagulation syndrome [20]. However, post-ESD infections have been reported. Chen et al. reported an infection rate of 12.2% following gastric ESDs for bulky stromal tumors [21], while Zeng et al. reported a rate of 16.3% in the case of esophageal mucosal lesions [22]. For EMR, the rate of post-procedural infections varies depending on the use of antibiotic prophylaxis up to 15.2% [23].

## 3. Mechanisms of Infection Transmission During Endoscopic Procedures

There are two main mechanisms of infection transmission during GI endoscopic procedures: (1) endogenous transmission, involving pathogens that colonize the patient or are part of an ongoing infection and can be translocated to other anatomical sites during the procedure, potentially leading to infections, and (2) exogenous transmission, which refers to the inoculation of microorganisms introduced via contaminated endoscopes or their accessories [24].

### 3.1. Endoscope-Related Transmission

GI endoscopy procedures involve the use of reprocessable flexible endoscopes such as gastroscopes, duodenoscopes used in ERCP and EUS, and colonoscopes, all of which require complex reprocessing protocols. Their structure is intricate due to the presence of internal channels, external components such as the duodenoscope elevator mechanism, and other accessories that may affect optimal reprocessing. In addition to the challenges related to the complex design of the endoscope, there is also a risk of biofilm formation within the working channels, especially through microfissures in the internal lining that may occur with repeated use. Contamination of flexible endoscopes is one of the main processes leading to healthcare-acquired infections caused by MDR bacteria and fungi, particularly in patients with immunosuppression. A thorough understanding of the pathophysiological mechanisms and reprocessing methods of flexible endoscopes is essential in order to reduce infection rates associated with GI endoscopic procedures [25]. Contamination rates of ready-to-use endoscopes are as high as 21.1%, according to a 2023 study. This represents an unacceptable risk to patients and highlights the need for additional measures to reduce endoscope contamination, optimize reprocessing protocols, and enhance microbiological surveillance [26].

In clinical practice, differentiating between endogenous and exogenous infections is often challenging. Infections caused by antibiotic-susceptible bacteria are frequently attributed to an endogenous mechanism, although they may also originate from contaminated devices, particularly duodenoscopes. In contrast, infections caused by MDR bacteria are less commonly reported despite being typically exogenous. This ambiguity contributes to the underestimation of the true incidence of post-endoscopic infections and hinders the accurate identification of their source. A study by Kwakman et al. estimated the risk of duodenoscope-associated infection (DAI) to be 0.01% per ERCP procedure, a value approximately 180 times higher than previously reported [27]. A systematic review published in 2022 reported an overall incidence of post-endoscopic infections of 0.2%, with variations depending on the type of procedure: 0.8% for ERCP, 0.123% for upper GI endoscopy, and 0.073% for lower GI endoscopy. The most frequently implicated pathogens were *Pseudomonas aeruginosa*, *Enterobacter* spp., and Gram-positive cocci [7,28].

Contamination rates of endoscopes are significant, ranging from 7.7% to 34.6%, depending on the type of device. Duodenoscopes, whether used for ERCP or EUS, have reported contamination rates between 0.697% and 60%. Infections with MDR bacteria, such as *Klebsiella pneumoniae* and *Pseudomonas aeruginosa*, are predominantly of exogenous origin. The escalation of antimicrobial resistance, particularly in carbapenemase-producing *K. pneumoniae* strains, poses a major challenge, with incidence increasing from 1% in 2000 to 12% in 2010 [29].

### 3.2. Transmission via Healthcare Workers

Transmission of microorganisms from healthcare workers to patients represents a real risk during endoscopic procedures and is primarily associated with non-compliance with protective standards, particularly the improper use of personal protective equipment (PPE). Proper use of PPE provides bidirectional protection, reducing the risk of pathogen transmission both to the patient and to the healthcare worker [30,31].

Endoscopy unit personnel are at an increased risk of colonization or infection with pathogens such as *Helicobacter pylori*, which may be transmitted to patients in the absence of strict hygiene measures and consistent use of PPE. In addition, bloodborne viruses such as hepatitis B and C viruses (HBV, HCV), human immunodeficiency virus (HIV), as well as respiratory pathogens like influenza virus or *Mycobacterium tuberculosis*, can also be transmitted to patients during endoscopic procedures [32].

In addition to the risks posed to patients, healthcare workers themselves are also vulnerable to infections transmitted from patients. Documented cases of transmission include accidental needlestick injuries and conjunctival exposure to infectious secretions, particularly for bloodborne viruses [33].

*Clostridioides difficile* is another relevant pathogen in the context of nosocomial transmission, including within endoscopy units. The rate of hand contamination among healthcare workers with *C. difficile* ranges from 0% to 59%, depending on adherence to hand hygiene practices and the level of environmental contamination. Additional risk factors for *C. difficile* infection include prior use of antibiotics and proton pump inhibitors, both of which are commonly encountered in patients undergoing endoscopic procedures [34].

### 3.3. Patient-to-Patient Transmission

The American Society for Gastrointestinal Endoscopy (ASGE) classifies pathogen transmission from one patient to another as either endoscopic or non-endoscopic. Endoscopic transmission occurs as the result of inadequate reprocessing performed by healthcare personnel, while non-endoscopic transmission is the result of the improper use of drug vials or syringes [31].

The administration of intravenous anesthetics, proper disinfection of endoscopes, and adherence to hygiene protocols play a critical role in preventing the transmission of infections between patients. However, studies have shown that healthcare personnel tend to apply stricter disinfection measures when the patient is known to have a transmissible infectious disease such as HBV, HCV, HIV, or tuberculosis. This practice, observed for several decades, still persists today despite the fact that many patients are either unaware of their infectious status or choose not to disclose it due to fear of stigmatization. A study conducted by Cowen in 1993 highlighted this discrepancy: in 96% of endoscopy units, full disinfection was performed for patients known to have HIV or HBV, compared to only 55% for patients suspected of having infectious colitis [35].

Aseptic medication administration errors represent a significant risk, particularly in the context of screening colonoscopies in high-risk populations, such as individuals aged 50 to 59 years, the age group with the highest prevalence of HCV infection in the United States. A notable case reported in 2010 described the transmission of hepatitis viruses through the repeated use of the same syringe to administer propofol to multiple patients. Contamination of a vial intended for single-patient use resulted in infections in 12 patients: 6 with HBV, 5 with HCV, and 1 with HBV/HCV coinfection. No HIV infections were reported in this context [36].

## 4. Infectious Agents Involved

### 4.1. Bacteria

A retrospective study conducted between 2015 and 2017, which included 12,714 patients who underwent endoscopic procedures (upper GI endoscopy, colonoscopy, ERCP, and enteroscopy), reported an overall incidence of post-procedural bacteremia of 0.43%. The most frequently isolated bacteria were *Escherichia coli* (18.2%), *Enterococcus faecalis* (10.9%), *Pseudomonas aeruginosa* (7.3%), and *Klebsiella* spp. (5.5%) [37].

Up to 15% of duodenoscopes deemed adequately reprocessed and ready for use in ERCP or EUS may still be contaminated with bacteria carrying significant infectious potential. Among the most frequently identified pathogens are Gram-negative bacteria such as *Escherichia coli*, *Klebsiella pneumoniae*, *Enterobacteriaceae* spp., and *Pseudomonas aeruginosa*, as well as Gram-positive organisms including *Staphylococcus aureus*, β-hemolytic *Streptococcus*, and *Enterococcus* spp. Fungi have also been isolated, particularly in immunocompromised patients [38,39,40]. Other microorganisms associated with post-endoscopic infections include *Helicobacter pylori*, *Serratia marcescens*, *Clostridioides difficile*, *Salmonella* spp., *Stenotrophomonas maltophilia*, and *Acinetobacter* spp. [41,42].

The infectious risk varies depending on the type of procedure performed. In diagnostic upper GI endoscopy, the most frequently isolated bacteria are *Staphylococcus epidermidis* and *Streptococcus* spp. In contrast, interventional procedures at the same level carry a significantly higher risk, with bacteremia rates reaching up to 54%. These patients may develop rare but severe complications such as peritonitis, endocarditis, abscesses, or disseminated infections. Lower GI endoscopies are primarily associated with the isolation of *Enterococcus* spp., *Enterobacteriaceae* spp., and *Bacteroides* spp. In PEG, the most common infections are caused by *Staphylococcus aureus*, *Klebsiella* spp., *Enterobacter* spp., *Pseudomonas aeruginosa*, *Enterococcus* spp., and *Candida albicans*. In ERCP, particularly in patients with biliary stents, the literature highlights a potential pathogenic synergism between *Escherichia coli* and *Enterococcus* spp., contributing to an increased risk of infection [24].

The etiology of post-endoscopic infections is often multifactorial. The most important contributing factors include the improper reprocessing of endoscopes, the complex and difficult to clean design of certain devices (such as the duodenoscope elevator mechanism), as well as potential structural defects. In some cases, no clear cause can be identified, which complicates both the prevention and surveillance of these infections [43].

#### 4.1.1. *Klebsiella* spp.

Over the past decade, infections with *Klebsiella pneumoniae* have been increasingly reported, particularly in the context of exogenous transmission via contaminated duodenoscopes. This trend is further exacerbated by rising antibiotic resistance and the emergence of extended-spectrum beta-lactamase (ESBL)-producing or carbapenem-resistant strains, with major clinical implications in both developed and developing countries [44]. Approximately 6.1% of *Klebsiella pneumoniae* strains exhibit resistance to imipenem or meropenem, a mechanism that is often plasmid-mediated [45].

Even with strict adherence to HLD protocols, the use of automated endoscope reprocessors (AERs), and proper storage of endoscopes, infections or asymptomatic colonization cannot be entirely eliminated. Major risk factors for invasive infections include the use of duodenoscopes (both for ERCP and EUS), upper GI endoscopic procedures, and a history of cholangiocarcinoma [45,46,47].

A study conducted by Humphries et al. identified nine cases of infection and seven asymptomatic carriers of carbapenem-resistant *Klebsiella pneumoniae* following ERCP procedures. A genomic analysis revealed the presence of the *bla_OXA-232_* gene, which is associated with carbapenem resistance [47].

Alrabaa et al. also reported seven cases of carbapenem-resistant *Klebsiella pneumoniae* infections occurring up to 60 days after ERCP procedures. Epidemiologic investigations identified inadequate duodenoscope reprocessing, particularly involving the elevator mechanism, as the likely source [48].

Cimen et al. documented the transmission of a *K. pneumoniae* strain carrying the *bla_SHV-12_* gene from a colonized patient to two others via a seemingly clean duodenoscope. Initial cultures taken from the endoscope were negative, but dismantling of the device revealed persistent contamination at the level of the forceps elevator [49].

In a case reported in Colombia, three post-ERCP infections with carbapenem-resistant *Klebsiella pneumoniae* led to the implementation of additional quality control measures for reprocessing, including adenosine triphosphate (ATP) bioluminescence testing. Elevated ATP levels indicated the need to repeat the cleaning process of the endoscopes, thereby contributing to the prevention of further transmissions [50].

#### 4.1.2. *Pseudomonas aeruginosa*

Similarly to other bacteria involved in post-endoscopic infections, *Pseudomonas aeruginosa* has been frequently associated with the use of duodenoscopes, mainly due to their complex technical design and the challenges of effective reprocessing [51,52]. These instruments contain multiple hard-to-reach components that favor microbial retention and compromise thorough cleaning, especially when strict cleaning protocols are not rigorously followed.

Verfaillie et al. reported an outbreak of infections caused by VIM-2-producing *Pseudomonas aeruginosa* following duodenoscope contamination. The epidemiological investigation required disassembly of the device, revealing that the design of the distal tip hindered effective reprocessing, and the O-ring failed to provide proper sealing at the forceps elevator, thereby facilitating bacterial accumulation and transmission [53].

In another study, Reiner et al. identified three cases of *Pseudomonas aeruginosa* infection following ERCP procedures. Microbiological analyses detected the pathogen in six out of the twelve duodenoscopes used in that unit. The most frequently contaminated areas were the elevator channel (45% of cases), the biopsy channel (27%), the water/air channels (18%), and the suction channel (9%). Surprisingly, *P. aeruginosa* was also isolated from the enzymatic solutions used for initial post-procedural rinsing, indicating potential deficiencies in the decontamination and storage process [54].

Bajolet et al. also reported four cases of *Pseudomonas aeruginosa* infection with ESBL-producing strains following upper GI endoscopy, two of which involved biopsy sampling. The epidemiological investigation revealed several non-compliances in the reprocessing workflow, including insufficient manual cleaning (less than the recommended 10 min), use of inappropriate brushes for channel calibration, disinfection instead of sterilization of suction cylinders, and incomplete drying of endoscopes. Additionally, structural defects were reported in the endoscope implicated in the infection transmission, highlighting the critical need for rigorous monitoring of equipment integrity [55].

#### 4.1.3. *Escherichia coli*

*Escherichia coli* infections associated with endoscopic procedures, particularly ERCP, can lead to significant mortality [56]. An outbreak reported by Wendorf et al., caused by AmpC-producing and carbapenem-resistant (CR) strains of *E. coli*, showed marked differences in clinical outcome: the mortality rate was 56% for CR *E. coli* and 9% for AmpC-producing *E. coli* [57].

A major outbreak reported in 2014 involved 39 cases of infection with carbapenem-resistant *Escherichia coli* producing New Delhi metallo-β-lactamase (NDM) following ERCP procedures. In this study, no reprocessing errors or structural defects of the duodenoscopes used were identified. However, as a preventive measure, the institution revised its disinfection protocol by replacing ortho-phthalaldehyde with ethylene oxide gas sterilization. After implementing this new protocol, no additional infections were reported [58].

The persistence of endoscopy-associated infections remains a major concern despite technological advances and the introduction of new endoscope designs. In 2024, eight cases of infection or colonization with *Escherichia coli* producing New Delhi metallo-β-lactamase (NDM) were reported in association with the use of gastroscopes and duodenoscopes. The epidemiological investigation, which included whole-genome sequencing (WGS), did not identify any reprocessing errors or contamination of the endoscopes, as cultures tested negative for the implicated strain. However, after the removal of the suspect gastroscopes and the introduction of duodenoscopes with disposable distal caps, no further cases of infection or colonization were reported [59].

#### 4.1.4. *Enterococcus* spp.

Post-endoscopic infections with *Enterococcus* spp. are frequently reported, particularly in the context of ERCP procedures. In cases of acute cholangitis, *Enterococcus* spp. has been identified in approximately 23.6% of cases; however, the incidence may rise to 43.6% in patients with a history of sphincterotomy or biliary stenting, suggesting an increased infectious risk in these subgroups [60].

A study conducted by Kühl et al. on a cohort of liver transplant recipients reported a post-ERCP infection rate with *Enterococcus faecium* of up to 30%, highlighting the particular vulnerability of certain patient categories during GI endoscopic procedures [61].

Although rare, post-endoscopic infective endocarditis has been reported in the literature, particularly in association with interventional endoscopic procedures. One documented case describes a 71-year-old patient with known mitral regurgitation who developed infective endocarditis caused by *Enterococcus faecalis* six weeks after undergoing endoscopic resection of a large polyp in the transverse colon. The patient had not received antibiotic prophylaxis for the endoscopic procedure, in accordance with current guidelines; however, he had previously been advised to receive prophylaxis for dental procedures [62]. Remarkably, cases of infective endocarditis caused by *Enterococcus faecalis* following a colonoscopy with polypectomy have also been reported in patients without structural valvular abnormalities, suggesting that procedure-induced transient bacteremia may have serious consequences even in the absence of evident cardiovascular risk factors [63].

#### 4.1.5. *Helicobacter pylori*

*Helicobacter pylori* transmission can occur both through healthcare and auxiliary personnel as well as during upper GI endoscopy, as previously discussed. Therefore, the use of personal protective equipment (PPE) by medical staff is essential during endoscopic procedures. In low- and middle-income countries (LMICs), the water used for rinsing endoscopes may be contaminated with fecal matter from infected patients, representing another potential route of transmission [64,65].

#### 4.1.6. *Clostridioides difficile*

Post-endoscopic *Clostridioides difficile* infections are rarely reported but may occur in particular clinical settings. The infection has been largely attributed to disruption of the normal gut microbiota following bowel preparation for colonoscopy, particularly in the absence of other overt risk factors [66]. Other potential mechanisms involved include contamination of the endoscope used during the procedure, as well as the administration of antibiotics, either for prophylactic or therapeutic purposes. However, in the general population, the absolute risk of developing *C. difficile* infection following an endoscopic procedure is considered to be very low [67].

Special attention should be given to patients with inflammatory bowel disease (IBD), in whom the risk of developing *C. difficile* infection after colonoscopy is significantly increased up to 13 times higher compared to patients without IBD. This increased vulnerability may be explained by the underlying intestinal inflammatory status, frequent use of immunosuppressive therapy, and preexisting microbial imbalances [68,69].

### 4.2. Viral Hepatitis and Other Viruses

Transmission of viruses through endoscopes is considered a rare but possible event, particularly in the context of inadequate reprocessing. Studies have shown that although the use of endoscopes in patients with HBV can result in contamination of working channels with viral DNA, standard HLD methods are generally effective in eliminating it, even under suboptimal conditions [70]. The difficulty in investigating and demonstrating post-endoscopic viral transmission is related to the viral incubation period, the loss of patients from follow-up, and the difficulty of phylogenetic analysis [71]. Nevertheless, rare cases have been reported. As early as 1983, a case was described in which a patient with cirrhosis and GI bleeding, who was in the incubation period of HBV infection, transmitted the virus via an endoscope that was used 24 h later in another female patient, who subsequently developed acute hepatitis B after 96 days [72].

Another study from 1978 showed that, although radiolabeled hepatitis B surface antigen (HBsAg) was detectable after routine reprocessing, none of the patients exposed to the same endoscope developed the infection [73]. Moreover, a large study involving 623 endoscopic procedures performed in areas with high HBV prevalence concluded that the risk of transmission is extremely low [74]. Additionally, another study involving 10 patients exposed to the same endoscope previously used for patients positive for both HBsAg and HBeAg did not identify any post-procedural infections [75].

Recently, Liu et al. reported a 10.4% contamination rate of endoscopes with HBV immediately after variceal ligation in infected patients. However, viral DNA was no longer detectable following standard cleaning and HLD [76]. In another study, 5042 patients who underwent endoscopy between 2002 and 2011 and were considered at risk for HBV, HCV, or HIV infection due to a reprocessing error did not develop any of these viral infections [77]. The main identified risk was associated with the reuse of instruments such as cytology brushes or biopsy forceps [78]. Overall, the risk of HBV transmission through endoscopy is considered extremely low, even in the absence of advanced reprocessing [79].

Regarding HCV, the most common modes of transmission are associated with hospitalization, breaches in aseptic technique, or the improper use of medication vials and syringes for injectable drug administration [80,81]. The estimated risk of HCV transmission following endoscopy is 1 in 10 million procedures [33]. Studies have shown that when reprocessing protocols are properly followed, no HCV transmission has been documented [33,82]. However, complex epidemiological investigations have identified probable cases of transmission, some of which may have been overestimated due to the co-existence of alternative transmission routes [36,83,84,85]. Prospective studies conducted in Italy [86] and Egypt [87] did not demonstrate HCV transmission when endoscopes were properly reprocessed, even if previously used for patients with HCV infection. However, other research suggests that biopsy and other invasive procedures may be associated with a viral transmission risk [88], although this risk remains lower compared to other diagnostic or therapeutic methods [89].

Regarding HIV, no cases of transmission through GI endoscopy have been reported, although it is theoretically possible in the presence of a defective working channel and improper reprocessing. The highest risk of HIV transmission has been associated with the reuse of disposable syringes for anesthesia administration [90,91,92,93,94,95,96]. The use of 2% glutaraldehyde during the pre-cleaning phase appears to be effective in eliminating the virus, which may explain the absence of HIV transmission via endoscopy [91].

### 4.3. Prions

Prion diseases are rare but fatal neurodegenerative conditions caused by abnormal prion proteins that undergo misfolding, resulting in pathological conformers resistant to cellular degradation. These proteins predominantly accumulate in the central nervous system but can also be identified in the small intestine, particularly in Peyer’s patches, as well as in the spleen, tonsils, and appendix [97]. The highest concentration of prions is found in the brain and tonsils, while the lowest levels are present in the rectal area [98].

Although no confirmed cases of prion disease transmission through GI endoscopy have been reported to date, major societies such as the European Society of Gastrointestinal Endoscopy (ESGE) and the American Society for Gastrointestinal Endoscopy (ASGE) have issued strict recommendations regarding preventive measures in this context [31,99]. It is emphasized that endoscopic procedures in patients with a confirmed diagnosis or high suspicion of prion disease should only be performed when absolutely indicated and when non-invasive methods fail to provide a satisfactory diagnosis. In such cases, the endoscope used should either be quarantined and reserved exclusively for future use in patients with confirmed or suspected prion disease, or it should be a device at the end of its operational lifespan, intended for subsequent disposal [31,99]. In contrast, for non-invasive diagnostic procedures (without biopsies, cytology brushing, hemoclip placement, or variceal injection), destruction or quarantine of the endoscope is not considered necessary [100].

Esophageal intubation should be performed with utmost care under direct visual control by an experienced endoscopist in order to avoid injury to the tonsils, which, as previously mentioned, may serve as a potential reservoir for prions. If tissue sampling is absolutely necessary, biopsies should be obtained using single-use forceps, and the working channel of the endoscope should be cleaned between each pass to reduce the risk of subsequent contamination [99].

Concerning reprocessing, it is recommended to avoid the direct application of glutaraldehyde-based agents on contaminated endoscopes before mechanical cleaning and prior rinsing, as glutaraldehyde has protein-fixing properties, including for prions, which may promote their adherence to the internal surfaces of the endoscope [101]. Instead, gaseous agents and combinations of detergents and proteolytic enzymes are recommended, as they have proven to be more effective in reducing the infectious prion load [100].

Modern automated endoscope reprocessing technology plays an important role in reducing prion protein contamination, ensuring superior decontamination compared to manual methods [101].

### 4.4. Fungi

Fungal infections associated with GI endoscopic procedures are rare, owing to the overall effectiveness of standard reprocessing protocols, which successfully eliminate most fungi that may contaminate endoscopes [95]. Nevertheless, isolated cases have been reported in the literature, particularly in the context of biofilm formation on AERs, which may serve as a fungal reservoir with infectious potential [102]. One example is the report of two cases of esophagitis caused by *Trichosporon asahii* in immunocompetent patients, with the infections attributed to the use of contaminated biopsy forceps during endoscopic procedures [103]. Moreover, the use of duodenoscopes in ERCP procedures, devices with a complex design that is difficult to clean effectively, may contribute to persistent contamination with fungi such as *Candida* spp. In immunocompromised patients, such contamination can lead to severe infections, including fungal sepsis [104]. Another reported case involved a patient with chronic pancreatitis who developed a severe *Candida* spp. infection 12 days after undergoing an ERCP procedure [105]. Fiorini et al. also described a case of acute cholangitis following ERCP in a patient with choledocholithiasis, in which microbiological analysis of the nasobiliary drainage tube revealed colonization with *Candida glabrata*, *Candida albicans*, and methicillin-resistant *Staphylococcus aureus* (MRSA) [106]. Other reports include cases of post-ERCP sepsis caused by *Candida tropicalis* [107] and *Candida albicans* [108] in immunocompetent patients, highlighting that the risk of fungal infection is not exclusively confined to immunocompromised patients.

In conclusion, although post-endoscopic fungal infections are rare, they can have a significant clinical impact, particularly in cases of immunosuppression, when complex devices such as duodenoscopes are involved, or when fungal biofilms develop within reprocessing systems. This category of infection warrants close monitoring and a rigorous preventive approach in endoscopy centers.

### 4.5. Parasites

Regarding parasitic infections, available data are limited. To date, four cases of parasitic infection have been reported, involving patients diagnosed with esophagitis caused by *Strongyloides* spp., transmitted through a contaminated endoscope. Following this outbreak, the endoscopy unit implemented gas-based reprocessing of the endoscope, which proved effective and was not associated with any further secondary parasitic infections [109].

## 5. Risk Factors for Transmission of Infections After Endoscopy

Conditions associated with an increased risk of post-endoscopic infections include decompensated cirrhosis with upper GI bleeding, cirrhosis with large-volume ascites, immunosuppression due to malignancy or immunosuppressive therapy, peritoneal dialysis, and biliary obstructions in which complete drainage of the bile ducts cannot be ensured (e.g., cholangiocarcinoma, primary sclerosing cholangitis) [4,110]. Endoscopic procedures associated with a high risk of infection include cholangioscopy, pancreatoscopy, EUS-FNA/FNB of cystic lesions, and PEG placement [4].

Patients with a history of malignancy, chemotherapy, radiotherapy, or organ transplantation who are undergoing immunosuppressive treatment are at a significantly increased risk of developing post-endoscopic infections, often caused by MDR organisms. A 2013 study from the United States reported three cases of infection with carbapenemase-producing Enterobacteriaceae in patients who underwent ERCP using the same duodenoscope. The index patient, a 57-year-old man, underwent ERCP for acute cholangitis due to choledocholithiasis; the second patient, aged 72, developed urinary sepsis two months after ERCP; and the third, a 23-year-old, experienced sepsis with the same pathogen six months post-procedure. All patients had underlying oncologic, hematologic, or autoimmune conditions known to be associated with immunosuppression [111].

An infectious risk assessment should encompass all equipment that comes into direct or indirect contact with patients. Decristoforo et al. conducted microbiological testing on reprocessed endoscopes and AERs, reporting contamination rates ranging from 1.3% to 4.6%. The most frequently isolated microorganisms were *Pseudomonas* spp., particularly *Pseudomonas oleovorans*, known for its biofilm-forming capacity within endoscope channels and AER components, posing the risk of invasive infections in immunocompromised patients. *Streptococcus* spp. was the second most frequently identified pathogen in the study. Although the overall contamination rate of endoscopes was low, the isolation of pathogenic bacteria such as *Staphylococcus aureus* and *Pseudomonas aeruginosa* still represents a significant infection risk, even for immunocompetent individuals [112].

A study conducted in Taiwan on 112,543 patients assessed the 30-day incidence and risk of infection following diagnostic colonoscopy, sigmoidoscopy, biopsy, or polypectomy. The most frequently reported infections were diverticulitis (37.96%), peritonitis (25.55%), appendicitis (13.38%), osteomyelitis (7.79%), pyogenic liver abscess (5.60%), and septic pulmonary embolism (1.95%). The authors developed a predictive nomogram for infection risk, identifying significant variables such as advanced age, male sex, cirrhosis, alcoholic liver disease, cholangitis, cholecystitis, and chronic kidney disease, with cirrhosis having the strongest impact. Therefore, a 70-year-old patient with cirrhosis and chronic kidney disease should be considered at high risk for post-endoscopic infectious complications [113]. Data have also been published regarding the risk of peritoneal dialysis patients developing polymicrobial peritonitis following endoscopic procedures, particularly colonoscopy, with antibiotic prophylaxis being recommended. A relevant example is that of a dialysis patient who developed peritonitis with *E. coli*, *K. pneumoniae*, and *E. faecalis* 24 h after undergoing a polypectomy [114].

Control of post-endoscopic infectious risk involves optimizing techniques that increase diagnostic accuracy, such as the use of simethicone to disperse residual air bubbles and mucosal secretions. Simethicone is administered via the water or working channel of the endoscope. However, it is difficult to remove during reprocessing, potentially contributing to contamination due to incomplete drying and its carbohydrate content, which provides a substrate for bacterial growth and proliferation [115,116].

Monitoring the effectiveness of endoscope reprocessing should include the assessment of residual bioburden, preferably through rapid and cost-effective methods such as ATP testing [117,118]. An ATP level below 200 relative light units (RLUs) indicates adequate reprocessing. These tests are particularly useful in resource-limited settings and help identify procedural errors while supporting the training of personnel involved in cleaning endoscopic devices [119]. In the case of duodenoscopes, which have a complex design, ATP testing can be supplemented or replaced by triple adenylate nucleotide (A3) testing, which provides superior sensitivity for detecting bacterial contamination [120]. Reuken et al., in a retrospective study from 2017, found that cholangitis caused by MDR bacteria was associated with more pronounced cholestasis and lower leukocyte counts compared to cholangitis due to non-MDR pathogens. The strongest independent risk factor for MDR-related cholangitis identified in this study was prior biliary stenting [121].

**Figure 1:**
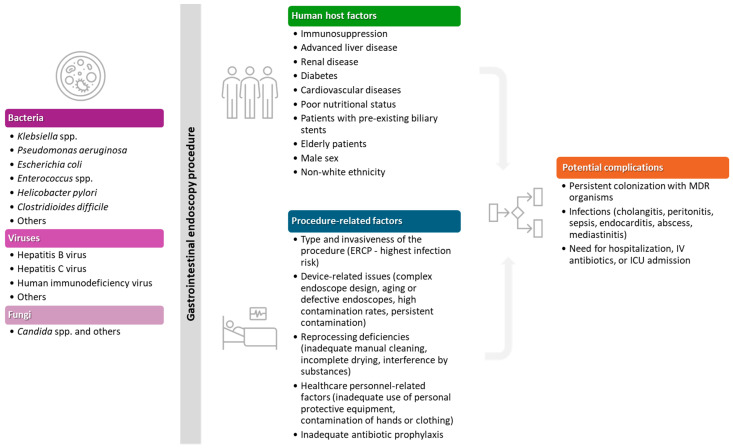
Overview of factors influencing infection risk in GI endoscopy. ERCP—endoscopic retrograde cholangiopancreatography; ICU—intensive care unit; IV—intravenous; and MDR—multidrug-resistant.

As illustrated in Figure 1, the risk of infection following GI endoscopy is the result of a complex interplay between the type of pathogen encountered, patient-specific susceptibility factors, and procedure-related variables such as reprocessing practices and operator technique. These elements ultimately shape the potential complications.

## 6. Preventive Measures for Endoscopy-Associated Infections

Factors contributing to endoscope contamination despite the use of HLD include device-related issues (such as aging endoscopes or defects in the working channel), human factors (including inadequate training or non-compliance with reprocessing guidelines), insufficient drying, and the use of substances that may interfere with the reprocessing cycle, such as simethicone or lubricants [122].

A retrospective study reported a contamination rate of up to 49% for endoscopes considered ‘ready for use’ after reprocessing [26].

The importance of pre-cleaning and the manual reprocessing of endoscopes must be emphasized, as these steps together reduce more than half of the total bacterial load [123]. The drying procedure of the endoscope channel plays a critical role, especially when reprocessing conditions are suboptimal and residual microorganisms persist in the fluid within the endoscope channel. Without complete drying, these pathogens can multiply and become a significant bacterial reservoir, posing a risk of infection to patients. Optimal drying can be achieved using medical-grade air compressors or dedicated endoscope drying cabinets [124]. Due to the variability between existing reprocessing guidelines, the amount of residual fluid inside the endoscope can range between 42% and 95%. Automated drying methods that apply forced filtered air for 5 or 10 min have demonstrated superiority over manual air-drying techniques. Although endoscope reprocessing has improved in recent years, there are still many process-related variables for which no clear consensus exists among current guidelines [125].

Several aspects of the endoscope reprocessing workflow still lack international consensus. These include the role of alcohol flushing and the type, quality, and duration of air flow, as well as the optimal drying time required before immediate use following HLD. Additionally, factors such as the storage cabinet conditions, vertical versus horizontal positioning of the endoscopes, the maximum storage interval before repeat reprocessing is required, and whether accessories should be stored together or separately from the endoscopes remain open questions in need of standardization [126].

Flushing endoscope channels with alcohol at concentrations of 30–50% can accelerate the drying process and reduce the risk of *Pseudomonas aeruginosa* contamination during storage [127], but it is not recommended in regions endemic for prion infections because of the protein-fixing properties of alcohol [126].

The literature data indicate that both the 3 min air and alcohol-drying cycle and the 10 min air-drying cycle available in AERs are insufficient for the complete removal of moisture from narrow channels (e.g., air/water channels). In contrast, these methods are effective for larger-diameter channels, such as the biopsy channel. These findings highlight the need to optimize and individualize the drying process, even when using AERs [128].

The water used for rinsing the endoscope and its channels must be properly filtered. Most post-endoscopic infections are associated with ERCP and EUS procedures, which commonly utilize duodenoscopes. The most challenging part to clean and reprocess is the duodenoscope’s elevator mechanism, which remains difficult to adequately disinfect even when using AERs. Therefore, manual cleaning remains essential for the effective decontamination of both the elevator and the working channel [129].

The steps of endoscope reprocessing may vary depending on the availability of AERs within the endoscopy unit [130]. However, the main stages of reprocessing follow a standardized sequence: (1) pre-cleaning, which must be performed immediately after the endoscopic procedure at the patient’s bedside, (2) manual cleaning, (3) HLD (manual or automated), and (4) storage [130,131]. When an AER is used, steps 2 and 3 are performed within the device [130]. Although the use of AERs is becoming increasingly widespread, current evidence does not demonstrate clear advantages over manual HLD in terms of reprocessing efficacy [132]. Actively moving the elevator mechanism up and down at least three times during the immersion phase of duodenoscope reprocessing, as well as the use of special endcaps, helps to reduce the bacterial load [130].

The Spaulding classification [133] categorizes reusable medical equipment based on the level of disinfection or sterilization required: critical devices (penetrating sterile tissue—requiring full sterilization), semi-critical (in contact with mucous membranes or damaged skin—requiring HLD), and non-critical (in contact with intact skin—requiring low-level disinfection). The appropriate chemical agents vary accordingly [134]. Given the high risk of infection transmission, duodenoscopes have been reclassified from semi-critical to critical devices, necessitating enhanced reprocessing protocols [135].

Conducting routine microbiological surveillance cultures of endoscopes can serve as an effective tool for monitoring the adequacy of reprocessing and for detecting potential breaches or errors in the decontamination process [136]. A 17-year study conducted in France demonstrated the positive impact of microbiological surveillance of reprocessed endoscopes, with contamination rates decreasing from 19.7% in 2004 to 13% in 2021 [26]. Similarly, a prospective study in Italy reported a contamination rate of 36.81% in reprocessed and stored duodenoscopes. These findings support the idea that microbiological surveillance represents a cost-effective, environmentally sustainable, and efficient strategy to reduce infection risks associated with GI endoscopy, potentially offering a viable alternative to the routine use of disposable duodenoscopes [137]. Regarding the frequency of the microbiological testing of endoscopes, current guidelines vary considerably, ranging from monthly intervals, particularly for duodenoscope surveillance, to every three months or even yearly intervals [130,138].

An emerging innovative approach to enhance bacterial eradication during endoscope reprocessing involves the use of side-emitting ultraviolet type C (UV-C) fiber optics, which can be inserted into the internal channels of the endoscope. This method offers significant advantages, such as low maintenance costs due to the lack of consumables, and has shown promising bacteriological efficacy in experimental models. However, it remains at the experimental stage and has not yet been incorporated into international reprocessing guidelines [139].

### 6.1. Antibiotic Prophylaxis in Infection Prevention

ESGE generally does not recommend routine antibiotic prophylaxis prior to ERCP. However, antibiotic prophylaxis before ERCP is suggested in the following situations: patients in whom complete biliary drainage is not anticipated (e.g., in primary sclerosing cholangitis or hilar obstructions), immunocompromised patients, and cases involving cholangioscopy [140].

Intraductal antibiotic prophylaxis administered concurrently with a contrast medium injection has been evaluated in several studies. One randomized controlled trial found no significant difference between gentamicin and distilled water injection (both mixed with the contrast medium) during ERCP performed for malignant biliary obstructions, mainly cholangiocarcinoma. All patients also received systemic antibiotic therapy, and clinical follow-up was conducted for up to 72 h post-procedure [141]. In contrast, a retrospective study demonstrated that adding a combination of vancomycin, gentamicin, and fluconazole to the contrast medium used during ERCP reduced the incidence of post-procedural infections from 33.3% to 14.3%, particularly in patients with primary sclerosing cholangitis and malignant biliary obstructions [142].

Regarding antibiotic selection, ESGE recommends adaptation to local epidemiology and the use of an agent active on Gram-negative pathogens [140]. Kühl et al. analyzed usage patterns in immunosuppressed patients, including liver transplant recipients, and reported the following distribution: piperacillin-tazobactam (73.7%), ceftriaxone (12%), meropenem (5.3%), ciprofloxacin (3.5%), and other antibiotics (5.5%) [61].

In patients with advanced chronic liver disease (ACLD) presenting with variceal upper GI bleeding, ESGE recommends prompt antibiotic prophylaxis, preferably ceftriaxone, for up to 7 days. However, some studies suggest that a 3-day course may be sufficient [143].

Similarly to ESGE, the ASGE guidelines recommend antibiotic prophylaxis in patients undergoing ERCP for biliary obstruction in the absence of clinical cholangitis, only when complete biliary drainage is unlikely to be achieved [144].

For EUS procedures, the ASGE advises antibiotic prophylaxis when performing an FNA of pancreatic or mediastinal cysts. Additionally, prophylaxis is indicated for all patients undergoing PEG placement, as well as for patients with cirrhosis presenting with acute upper GI bleeding starting from admission, regardless of the planned endoscopic procedure. Patients on peritoneal dialysis undergoing colonoscopy are also suggested to receive prophylaxis to prevent bacterial peritonitis [144]. Recent evidence, including the 2025 ESGE guidelines, no longer recommends antibiotic prophylaxis for the EUS-guided FNA of pancreatic cystic lesions [145].

Regarding the prevention of infective endocarditis, the ASGE guidelines recommend antibiotic prophylaxis only in patients with high-risk cardiac conditions when a GI infection involving *Enterococcus* spp. is present [144]. The European Society of Cardiology has recently upgraded its recommendation for endoscopic prophylaxis in high-risk patients from Class III (not recommended) to Class IIb (may be considered), despite the lack of evidence from randomized controlled trials. High-risk categories include individuals with ventricular assist devices, complex congenital heart disease, prosthetic valves (either surgical or transcatheter), prosthetic valvular material, or a history of infective endocarditis [146]. Considering the cases of endocarditis reported in the literature after colonoscopy with EMR in patients with native valves [62,63], and in the absence of clear guideline recommendations, we consider it necessary to individualize antibiotic prophylaxis in patients scheduled to undergo such procedures [147].

According to the Endoscopy Committee of the British Society of Gastroenterology, antibiotic prophylaxis is recommended in the following situations: ERCP without complete biliary drainage, FNA or endoscopic drainage of pancreatic cysts/pseudocysts, biliary complications following liver transplantation, PEG placement, variceal upper GI bleeding, and in immunocompromised patients with severe neutropenia or hematologic malignancies undergoing high-risk endoscopic procedures. While prophylaxis is unanimously supported for these patient groups, it is not recommended solely on the basis of increased cardiovascular risk for infective endocarditis in patients undergoing ERCP [148]. Recommendations for antibiotic prophylaxis in GI endoscopic procedures are summarized in Table 1.

### 6.2. Hygiene Practices for Healthcare Workers

In most endoscopic procedures, the use of anesthesia has become a standard practice that enhances both patient and endoscopist comfort. However, the administration of anesthetic agents involves invasive techniques such as peripheral venous catheterization, which breach natural protective barriers and increase the risk of infection. The intravenous administration of analgesics, anesthetics, or fluids must be performed under strict aseptic conditions in accordance with international infection control standards [150,151].

Reusing syringes or administering intravenous medication from the same syringe to multiple patients is strictly prohibited. The use of single-dose vials is strongly recommended for each individual patient. If multi-dose vials are used, they must be accessed with a new sterile disposable needle or cannula for each patient to prevent cross-contamination [152].

A notable example highlighting the consequences of non-compliance with these safety practices occurred in Las Vegas in 2007, when six individuals contracted HCV following endoscopic procedures. The outbreak was traced to the reuse of disposable syringes and vials for administering intravenous medication to multiple patients. As a result, over 40,000 patients from the implicated clinic were notified about the potential risk of HCV exposure [153].

## 7. Diagnosis and Management of Infections Associated with Interventional Endoscopy

Clinical signs of infection associated with GI endoscopic procedures may include fever, chills, abdominal pain, dyspnea, urinary symptoms, altered general condition or mental status, and, in severe cases, signs of septic shock. These manifestations typically occur within 30 days following the procedure [154].

EUS-FNA carries a significant risk of mediastinitis, particularly in patients undergoing the procedure for cystic mediastinal lesions or necrotic mediastinal lymphadenopathy [155]. Beyond EUS, it is important to emphasize that any endoscopic procedure involving esophageal intubation carries a risk of esophageal perforation, a well-recognized classic cause of mediastinitis. The clinical presentation typically includes, in decreasing order of frequency: dysphagia; retrosternal pain radiating to the interscapular region, neck, or shoulders; nausea and vomiting; fever and chills; dyspnea; and confusion [156].

The Charcot triad (comprising fever, jaundice, and right upper quadrant abdominal pain) has a high specificity but low sensitivity for diagnosing acute cholangitis. In other words, while the presence of all three signs strongly supports the diagnosis, many cases may not present with the full triad. According to the 2018 update of the Tokyo Guidelines for the management of acute cholangitis [157], the Charcot triad is no longer considered a reliable diagnostic criterion and is not included in the formal diagnostic algorithm. The diagnosis of acute cholangitis is guided by the Tokyo Guidelines, which classify diagnostic criteria into three categories: Category A includes clinical signs of systemic inflammation such as fever or chills or laboratory evidence of inflammation (e.g., C-reactive protein > 5 mg/dL, leukocyte count < 4000/mm^3^ or >12,000/mm^3^). Category B refers to cholestasis, evidenced by elevated bilirubin or liver enzymes (e.g., AST, ALT, ALP, or GGT). Category C includes imaging findings suggestive of biliary obstruction, such as bile duct dilatation or the presence of an obstructive lesion.

A diagnosis of suspected cholangitis requires at least one item from category A in combination with one item from either category B or C. A definite diagnosis requires the presence of at least one item from each of the three categories [157]. In both suspected and confirmed cases of acute cholangitis, a fever exceeding 38 °C is a key clinical criterion. When cholangitis occurs after ERCP, the fever typically develops within 24 to 48 h post-procedure and must not have been present prior to the intervention. Identified risk factors for post-ERCP cholangitis include age over 60 years, the presence of hilar biliary obstructions, a prior history of ERCP, and previous episodes of cholangitis. Conversely, complete biliary drainage, particularly in the setting of choledocholithiasis, is considered a protective factor [158]. Miyatani et al. identified advanced age (≥75 years) and a main bile duct diameter of ≥12 mm as significant risk factors for the development of post-ERCP cholangitis [159].

As this is a healthcare-associated infection, the generally accepted risk window for post-ERCP infections ranges from 1 to 30 days after the procedure [154]. Diagnosis is based on the presence of a fever >38 °C, new-onset or worsening abdominal pain, changes in liver function tests, and the exclusion of other potential sources of infection such as aspiration pneumonia, cholecystitis, or pancreatitis [159,160,161].

Post-ERCP cholangitis can be classified into three categories: Mild cholangitis is defined by the onset of a fever ≥38 °C, lasting 24–48 h; moderate cholangitis is characterized by a febrile or septic state requiring hospitalization for more than 3 days or the need for endoscopic or surgical intervention; and severe cholangitis is marked by the development of septic shock or the necessity of surgical treatment. [162,163].

Patients undergoing PEG are frequently immunocompromised, often due to underlying malignancies and concurrent chemotherapy and/or radiotherapy. As a result, the risk of infection remains elevated, even when prophylactic antibiotics are administered. Infections can manifest early (within 7 days) or late (after 7 days), typically presenting with peristomal erythema and purulent discharge at the insertion site [164].

Patients undergoing GI endoscopic polypectomy may develop postprocedural fever, which can arise from several potential causes, including post-polypectomy coagulation syndrome or GI perforation. Jing et al. reported two cases of post-polypectomy infections following a colonoscopy, where patients presented with a high fever (39–40 °C) within hours to a few days after the procedure, in the absence of clinical or imaging evidence of GI perforation or other infectious sources. Clinical management in such cases may escalate to intensive care unit (ICU) admission due to the progression toward multiple organ dysfunction. The diagnostic workup should include a physical examination to exclude peritonitis, inflammatory, and sepsis markers (e.g., C-reactive protein, procalcitonin) and to obtain a complete blood count and contrast-enhanced CT imaging of the chest, abdomen, and pelvis [165].

### 7.1. Methods of Diagnosis

Diagnostic methods for post-ERCP cholangitis are diverse and include imaging modalities such as computed tomography (CT), magnetic resonance imaging (MRI), and magnetic resonance cholangiopancreatography (MRCP), as well as repeat ERCP with bile sampling for microbiological cultures to identify the causative pathogen and guide targeted antibiotic therapy [166]. Bile cultures demonstrate a higher diagnostic yield compared to blood cultures in post-ERCP cholangitis, making them the preferred method for identifying the causative pathogen [154,167]. Whenever positive, Gram and Giemsa smears should also be performed.

In general, the accurate identification and epidemiological linkage of post-ERCP infections requires concordant microbiological findings from bile, blood, and duodenoscope cultures, all isolating the same microorganism. Pulsed-field gel electrophoresis can be employed to determine clonal relatedness between isolates from patient samples and those obtained from endoscopic equipment [168].

Upper GI endoscopy carries the risk of esophageal perforation, a complication that can lead to mediastinitis. The clinical picture is often non-specific, with fever, chills, and tachycardia being common findings. Laboratory tests may reveal leukocytosis and elevated C-reactive protein and procalcitonin levels, while progression to sepsis can be accompanied by thrombocytopenia and disseminated intravascular coagulation. Diagnosis relies on imaging, with a contrast-enhanced CT of the neck and chest being the modality of choice. From a microbiological standpoint, esophageal perforation in previously healthy adults without recent antibiotic exposure typically yields *Streptococcus* spp., *Neisseria* spp., *Haemophilus* spp., and anaerobes. In contrast, patients with prior antibiotic therapy may harbor aerobic Gram-negative bacilli, *Staphylococcus aureus*, or *Candida* spp. [169].

Post-polypectomy infections may occur either as a result of transient bacteremia or as a complication of colonic perforation. These infections should be distinguished from post-polypectomy coagulation syndrome, which presents with a similar clinical presentation including abdominal pain, fever, chills, nausea, and vomiting, along with laboratory findings such as neutrophilic leukocytosis and an elevated C-reactive protein. The differential diagnosis relies primarily on CT to rule out pneumoperitoneum, which would suggest perforation [170]. Bacteremia following both diagnostic and therapeutic colonoscopy may lead to acute or subacute infective endocarditis in patients with underlying valvular heart disease [6]. In such cases, blood cultures should be obtained to identify the causative pathogen, alternative sources of infection should be ruled out, and transesophageal echocardiography should be performed to evaluate for endocardial involvement [146].

In the case of post-PEG infections, it is essential to obtain samples from the peristomal site for bacterial culture and antibiotic susceptibility testing. The most commonly isolated pathogens include *Staphylococcus aureus* and *Pseudomonas aeruginosa* [19].

### 7.2. Infection Prevention and Control; Infection Management

An effective strategy to prevent pathogen transmission during interventional endoscopy is the implementation of redesigned duodenoscope models in endoscopy units. The U.S. Food and Drug Administration (FDA) recommends replacing traditional duodenoscopes with fixed endcaps with models featuring disposable endcaps. This approach significantly reduces the risk of procedure-related infections, particularly in complex interventions such as ERCP [171].

#### 7.2.1. Management of Post-ERCP Infections

Management of post-ERCP cholangitis includes conservative, endoscopic, and surgical approaches, chosen based on the patient’s clinical status and the location and extent of the infection. Conservative therapy, consisting primarily of intravenous antibiotics and supportive care, represents the first-line treatment. In cases where conservative measures are unsuccessful, biliary decompression becomes necessary, typically through repeat ERCP with stent insertion or replacement or percutaneous transhepatic biliary drainage. Surgical intervention is reserved for refractory cases or when endoscopic and percutaneous methods are contraindicated or unsuccessful [172]. According to the Tokyo Guidelines, 2018, the severity of acute cholangitis is classified into three categories: mild, moderate, and severe. It is necessary that at least one of the following criteria is present for the diagnosis of severe acute cholangitis: hemodynamic instability with dopamine/norepinephrine requirements, neurological dysfunction, acute respiratory failure, renal failure, hepatic failure (International Normalized Ratio (INR) > 1.5), or hematologic dysfunction (Platelets (PLT) < 100.000/mm^3^) [157].

Haal et al. reported that a 3-day course of antibiotic therapy was sufficient in cases of acute cholangitis with complete biliary drainage achieved via ERCP. The empiric antibiotic regimens used in their study included: piperacillin/tazobactam (16.6%), ceftriaxone plus metronidazole (14.2%), amoxicillin/clavulanic acid (14.2%), ceftriaxone plus gentamicin (11.8%), ceftriaxone alone (11.8%), amoxicillin/clavulanic acid plus gentamicin (11.5%), and amoxicillin/clavulanic acid plus ciprofloxacin (10.8%) [173]. In cases of cholangitis complicated by liver abscesses, antibiotic therapy requires a prolonged duration. According to a study by Curran et al., the treatment duration ranged from 8.4 to 68.9 days [174]. In the literature, short-course antibiotic therapy of up to 2 days has been described as sufficient for patients with mild to moderate acute cholangitis, provided that complete biliary drainage was achieved by ERCP [175].

In a 2020 study by Kawamura et al., the most frequently used empiric antibiotic regimen was sulbactam/cefoperazone. In cases with prior sphincterotomy or biliary stent placement associated with a higher risk of *Enterococcus* spp. infection, penicillin was added. For patients with a history of ampicillin-resistant *Enterococcus* spp. infection, vancomycin was used in combination with sulbactam/cefoperazone [176]. Meropenem was considered in patients with previous bile cultures demonstrating susceptibility, while fluoroquinolones were alternatives for penicillin- or cephalosporin-allergic individuals [176].

Unfortunately, empirical antibiotic therapy is effective as a first-line treatment in only a limited number of cases due to the increasing prevalence of antimicrobial resistance [177]. Therefore, it is essential to ensure adequate biliary drainage endoscopically or radiologically, particularly in patients with incomplete initial drainage. This step is crucial to eliminate stagnant bile and contrast media, which can promote bacterial growth [178].

#### 7.2.2. Management of Post-EUS Infections

Antimicrobial therapy for infections associated with GI endoscopic procedures should be individualized based on clinical severity and the pharmacokinetic/pharmacodynamic properties of the antibiotics, including their ability to reach effective concentrations at the site of infection. Bacteremia associated with EUS procedures occurs in 0–5.8% of cases, although clinically apparent infections remain rare [17]. Identified risk factors for post-EUS infections include cystic lesions larger than 15 cm in diameter, the presence of acute necrotizing pancreatitis with collections that cannot be adequately drained using plastic stents, and the presence of pelvic cystic lesions [155,179]. Management of infections following EUS-FNA of pancreatic cystic lesions may begin with oral beta-lactam antibiotics. However, in cases of clinical deterioration or in patients with immunodepression, it is often necessary to escalate therapy to intravenous broad-spectrum agents, such as carbapenems [180].

#### 7.2.3. Management of Post-Polypectomy Infections

Mild to moderate infectious complications following colonoscopic polypectomy are typically managed with bowel rest, intravenous fluids, and broad-spectrum antibiotics [170]. In severe cases of post-polypectomy infection, marked by multiple organ dysfunction, admission to the ICU and initiation of broad-spectrum antimicrobial therapy targeting both aerobic and anaerobic pathogens is essential. In the case series reported by Jing et al., two patients with severe infections responded favorably to intravenous regimens: one was treated with meropenem (1 g every 12 h) and metronidazole (500 mg every 12 h), while the other received vancomycin (1 g every 12 h) in combination with imipenem/cilastatin (1 g every 8 h). Both patients showed clinical improvement and the resolution of symptoms following a relatively short course of antibiotics [165].

#### 7.2.4. Management of Post-PEG Infections

Management of post-PEG infections involves local antiseptic care of the peristomal site and the administration of broad-spectrum antibiotics. In early or mild cases, oral or enteral antibiotic therapy may be sufficient, while intravenous administration is required for severe infections. If there is no clinical improvement, treatment must be escalated. In cases complicated by peritonitis, abscess formation, or persistent infection, removal of the gastrostomy tube and surgical intervention may be necessary [181].

#### 7.2.5. Management of Post-Endoscopic Endocarditis

Although rare, infective endocarditis may develop as a complication following GI endoscopic procedures. Management requires the early initiation of targeted antimicrobial therapy, and, in severe cases, surgical intervention with replacement of the affected valve may be necessary [63].

According to the European Society of Cardiology (ESC) guidelines, the treatment of infective endocarditis should be individualized based on the causative pathogen and the type of valve involved (native vs. prosthetic). For *Enterococcus faecalis* infections, ampicillin plus gentamicin has historically been the mainstay of treatment; more recent data has shown that the combination of ampicillin and ceftriaxone can reduce the risk of nephrotoxicity by avoiding aminoglycoside use. The duration of therapy depends on the valve type: 4–6 weeks for native valve endocarditis (NVE) and more than 6 weeks for prosthetic valve endocarditis (PVE). Aminoglycosides are only recommended for a few days until clinical improvement and not more than 2 weeks. Additionally, rifampin and daptomycin may be considered for staphylococcal or enterococcal endocarditis, especially in resistant cases, often in combination with beta-lactams or fosfomycin [146].

## 8. Limitations and Future Directions

This review is intended to provide a current, comprehensive, and detailed picture of GI-associated infections; however, several limitations should be acknowledged. The cited references demonstrate substantial heterogeneity due to their worldwide origin. Reported infection rates vary considerably according to geographic region, type of endoscopic procedure, patient-specific factors, and the use of prophylactic antibiotic therapy, which differs from one center to another. Most of the included studies report a post-procedural follow-up limited to 30 days, which is not entirely adequate from an epidemiological perspective. In addition, some of the cited literature consists of case reports or outbreak-related case series, which do not provide a clear estimate of the global risk of these infections. Viral infections, although very rarely reported, have not been the subject of recent studies in the past years (2020–2025). Most contemporary studies focus instead on MDR bacterial infections and on novel approaches for endoscope reprocessing.

Regarding future perspectives, multicenter observational studies are needed to accurately document infections occurring after GI endoscopy, ideally extending the follow-up to 90 days, and supported by epidemiological investigations based on genetic sequencing (particularly for MDR bacteria). The recently updated guidelines on antibiotic prophylaxis in GI endoscopy should be implemented in most countries in order to assess their validity and real-world applicability. Furthermore, prospective studies evaluating new methods of endoscope reprocessing must continue at an accelerated pace in parallel with the development of novel duodenoscope models designed to overcome the shortcomings of current devices in terms of reprocessing. Prospective studies are also warranted in high-risk patient populations, with targeted periprocedural prophylactic strategies. Future research should also address in greater depth the mechanisms of infection transmission, as well as the implications, utility, cost, and environmental impact of single-use duodenoscopes. Finally, the diagnosis of GI endoscopy-associated infections should be individualized and incorporated into clear standardized diagnostic protocols for both clinicians and researchers.

## 9. Conclusions

While procedures such as diagnostic upper GI endoscopy or diagnostic colonoscopy carry low rates of infectious adverse events, others such as ERCP or PEG placement are associated with a higher risk. GI endoscopy-related infections may be caused by viruses, bacteria, prions, fungi, and parasites, with the vast majority of reports involving bacteria, with MDR bacteria being particularly problematic. Effective prevention requires a combination of rigorous reprocessing protocols, adherence to hygiene standards, judicious use of antibiotic prophylaxis, and the regular microbiological surveillance of equipment. Equally important is the early recognition and appropriate management of infections based on clinical presentation and procedure type. Future efforts must prioritize the development of optimal reprocessing technologies, the implementation of disposable endoscope components where appropriate, and the establishment of standardized diagnostic criteria for post-endoscopic infections. By integrating innovation, guideline adherence, and individualized risk assessment, clinicians can significantly reduce the burden of infection and improve the safety and outcomes of interventional GI endoscopy.

## Figures and Tables

**Table 1:** Recommendations for antibiotic prophylaxis in GI endoscopic procedures.

Recommended by	Type of Procedures	Types of Patients
European Society of Gastrointestinal Endoscopy [140,143,149]	ERCP ERCP with cholangioscopy	Incomplete biliary drainage anticipated Patients with severe immunosuppression
	Upper GI endoscopy	ACLD with acute variceal upper GI bleeding
	Esophageal dilation Variceal sclerotherapy Laser therapy in upper GI tract	High-risk cardiac conditions, vascular graft < 1 year, and severe neutropenia
	PEG	All patients
American Society for Gastrointestinal Endoscopy [144]	ERCP	Expected incomplete biliary drainage in absence of cholangitis
	EUS-FNA	Suggested for mediastinal, pancreatic or, peripancreatic cysts (Low quality of evidence)
	PEG	All patients
	All types of endoscopic procedures	Upper GI bleeding in patients with cirrhosis
	Colonoscopy	Peritoneal dialysis
	Interventional endoscopic procedures	High-risk cardiac patients with suspected enterococcal GI infection
British Society of Gastroenterology [148]	ERCP	Incomplete biliary drainage Primary sclerosing cholangitis or hilar cholangiocarcinoma Liver transplant history, pancreatic pseudocyst, severe neutropenia, or advanced hematologic malignancy
	PEG or PEJ	All patients
	EUS-FNA	Infected cysts and cystic lesions in/near pancreas; transgastric or transenteric drainage of pseudocysts
	Upper GI endoscopy	Acute GI bleeding in decompensated liver disease

Abbreviations: EUS-FNA—endoscopic ultrasound fine needle aspiration; ERCP—endoscopic retrograde cholangiopancreatography; ACLD—advanced chronic liver disease; GI—Gastrointestinal; PEG—percutaneous endoscopic gastrostomy; and PEJ—percutaneous endoscopic jejunostomy.

## Data Availability

No new data were created or analyzed in this study.
